# Supplementary material for: Selective blockade of Cav1.2 (α1C) versus Cav1.3 (α1D) L-type calcium channels by the black mamba toxin calciseptine
Source: Nat Commun. 2024 Jan 2;15:54. doi: 10.1038/s41467-023-43502-w (PMC10762068; doi:10.1038/s41467-023-43502-w)
Supplement: Supplementary file 1 — Supplementary Information [file 41467_2023_43502_MOESM1_ESM.pdf]

a

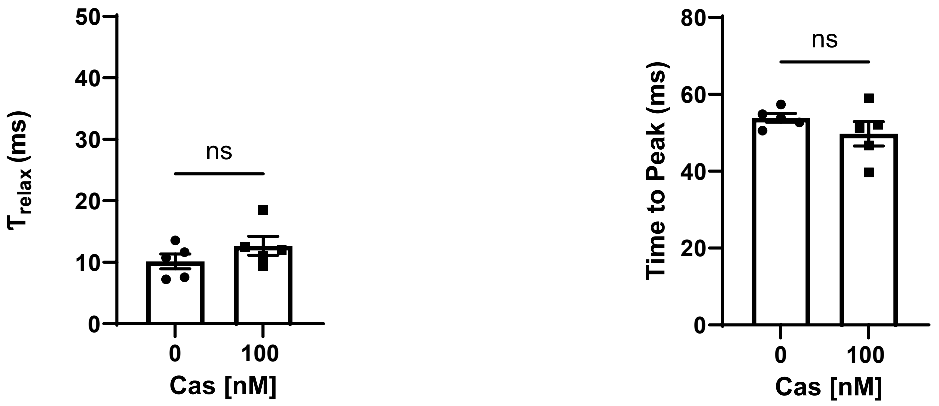

b

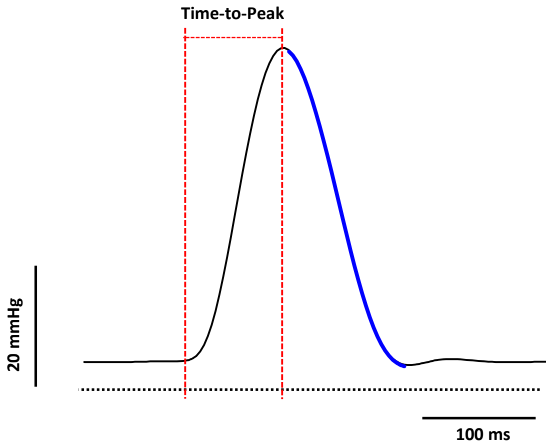

a. Time constant of LV isovolumic relaxation τ (left panel) and time-to-peak interval (right panel) before and after 100nM Cas perfusion (n=5). Statistics: paired two-sided Student t-test. Data are presented as mean values ± SEM. **b**. Representative sweep of a contraction event recorded in control condition. Time-to-peak (ms) is defined as the time interval between the point where the pressure signal starts to increase and the peak of the event. Blue curve represents the fitting of pressure signal during isovolumic left ventricular relaxation time with standard exponential function ($f(t)= Ae^{-t/\tau} + B$) to calculate τ. Dashed black line represent the zero pressure level.

Supplementary Fig.1

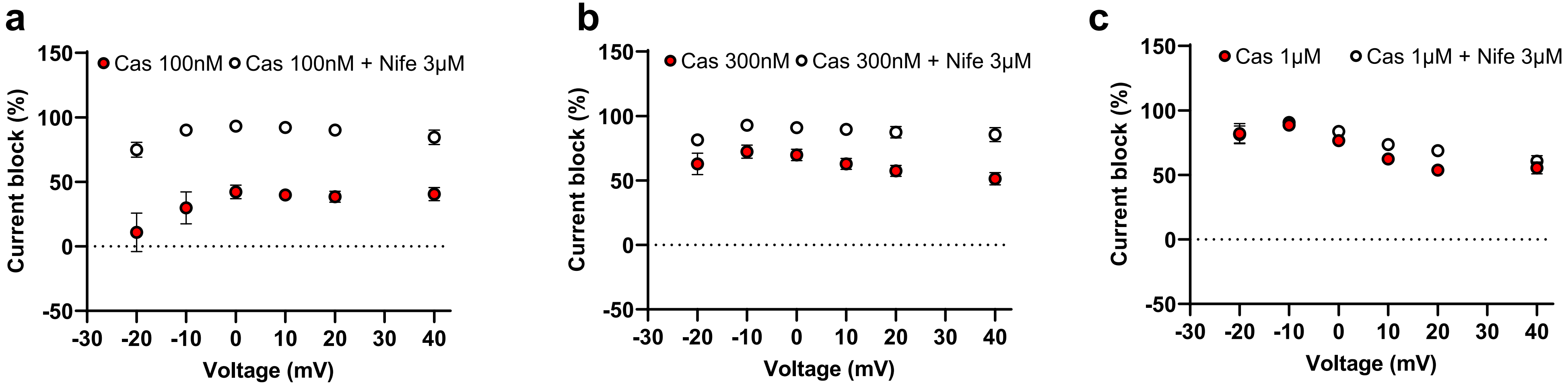

Percentage of $Ca_v1.2$ Ca^{2+} current blocked by Cas (n=9 100nM in a, n=7 300nM in b and n=6 1µM in c; red circles) or by Cas + 3µM Nifedipine (white circles) at membrane potential from -20mV to +40mV in isolated Wild-Type ventricular cells.

d

	Ctrl (pA/pF)	Cas 100nM (pA/pF)	Nife 3µM + Cas 100nM (pA/pF)
**-20mV**	-1.26±0.27	-1.21±0.20$^{\$}$	-0.29±0.02$^{\#}$
**-10mV**	-5.27±0.86	-3.45±0.54$^{\$\$}$	-0.36±0.04$^{\#\#}$
**0mV**	-9.40±0.95***	-3.45±0.54$^{\$\$}$	-0.59±0.10$^{\#\#\#}$
**+10mV**	-9.81±1.32***	-6.14±1.02$^{\$\$}$	-0.70±0.15$^{\#\#\#}$
**+20mV**	-8.04±1.33**	-5.17±0.93$^{\$\$\$}$	-0.71±0.21$^{\#\#\#\#}$
**+40mV**	-3.68±0.68*	-2.24±0.46$^{\$\$}$	-0.55±0.19$^{\#\#\#\#}$

e

	Ctrl (pA/pF)	Cas 300nM (pA/pF)	Nife 3µM + Cas 300nM (pA/pF)
**-20mV**	-2.02±0.86	-0.52±0.07$^{\$}$	-0.25±0.04$^{\#}$
**-10mV**	-6.58±1.29*	-1.63±0.37$^{\$}$	-0.26±0.02$^{\#}$
**0mV**	-10.51±1.51**	-3.07±0.59$^{\$}$	-0.70±0.11$^{\#\#}$
**+10mV**	-10.15±1.38**	-3.66±0.55$^{\$}$	-0.78±0.16$^{\#\#}$
**+20mV**	-8.09±1.11**	-3.32±0.45$^{\$\$}$	-0.71±0.15$^{\#\#}$
**+40mV**	-3.36±0.49**	-1.58±0.23$^{\$}$	-0.34±0.10$^{\#\#\#\#}$

f

	Ctrl (pA/pF)	Cas 1µM (pA/pF)	Nife 3µM + Cas 1µM (pA/pF)
**-20mV**	-3.53±0.98**	-0.34±0.07	-0.38±0.08$^{\#\#}$
**-10mV**	-7.71±0.98**	-0.87±0.23	-0.70±0.14$^{\#\#}$
**0mV**	-9.85±1.04****	-2.28±0.33	-1.57±0.20$^{\#\#\#\#}$
**+10mV**	-8.83±1,09***	-3.38±0.15	-2.41±0.09$^{\#\#\#\#}$
**+20mV**	-6.41±0.90**	-3.38±0.12	-2.40±0.12$^{\#\#\#}$
**+40mV**	-2.75±0.60*	-1.35±0.11	-1.19±0.10$^{\#}$

L-type Ca^{2+} current density at different voltages in isolated ventricular wild-type myocytes in control condition (Ctrl), after perfusion of Cas and after concomitant application of Nifedipine 3µM and Cas (100nM in d, 300nM in e and 1µM in f). *Control vs Cas, $^{\#}$Control vs Nife 3µM + Cas, $^{\$}$Cas vs Nife 3µM + Cas. Statistics: one-way ANOVA test followed by Tukey's multiple comparisons test. *p<0.05, **p<0.01, ***p<0.001, ****p<0.0001, $^{\#}$p<0.05, $^{\#\#}$p<0.01, $^{\#\#\#}$p<0.001, $^{\#\#\#\#}$p<0.0001, $^{\$}$p<0.05, $^{\$\$}$p<0.01, $^{\$\$\$}$p<0.01.

Supplementary Fig.2

a

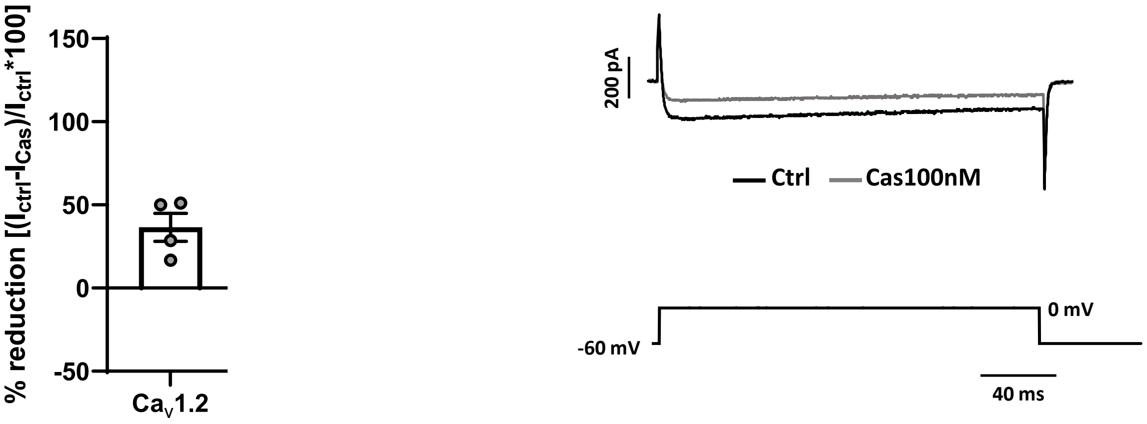

(Left panel). Histograms represent the percentage of reduction of L-type Ca^{2+}peak current density after 100nM Cas perfusion in HEK-293T cells transfected with $Ca_v1.2$ (n=4) channels. Peak current density was recorded starting from a holding potential of -60mV using an activating voltage step at 0mV. (Right panel). Representative traces of $Ca_v1.2$ L-type Ca^{2+}current before (black line) and after 100nM Cas (gray line) perfusion and recording protocol are showed. Data are presented as mean values ± SEM.

b

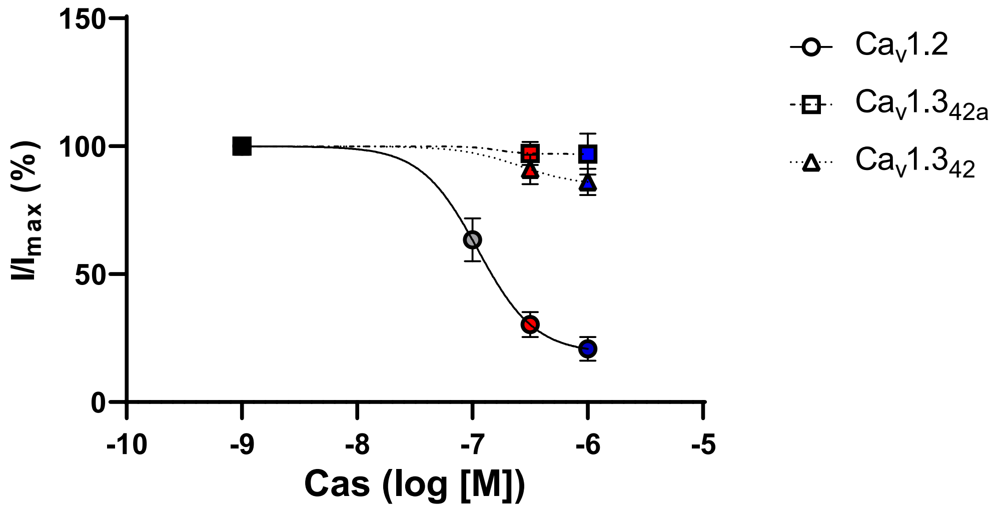

Concentration-dependent inhibition of $Ca_v1.2$ (n=11 in control condition, n=4 at 100nM, n=14 at 300nM n=7 at 1μM Cas; continuous line, circles), $Ca_v1.3_{42a}$ (n=9 in control condition, n=13 at 300nM n=8 at 1μM Cas dashed line, squares) and $Cav1.3_{42}$ (n=6 in control condition, n=6 at 300nM n=7 at 1μM Cas dotted line, triangles) channels by Cas in HEK-293T cells. Data are presented as mean values ± SEM.

Supplementary Fig.3

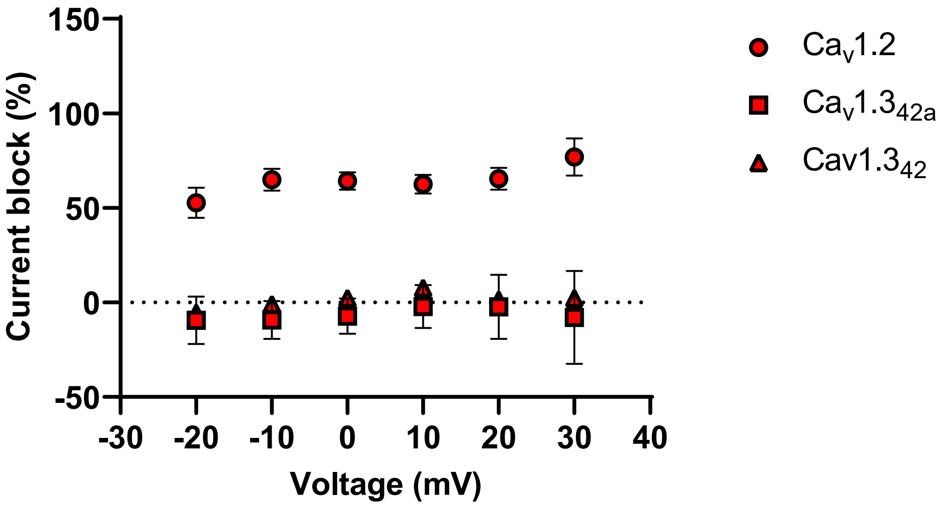

Percentage of current blocked by 300nM Cas at membrane potentials between -20mV and +30 mV in $Ca_v1.2$ (n=11, circles), $Ca_v1.3_{42a}$ (n=9, squares) and $Ca_v1.3_{42}$ (n=6, triangles) transfected HEK-293T cells. Data are presented as mean values ± SEM.

	**$Ca_v1.2$**		**$Ca_v1.3_{42a}$**		**$Ca_v1.3_{42}$**	
	**Ctrl (pA/pF)**	**Cas 300nM (pA/pF)**	**Ctrl (pA/pF)**	**Cas 300nM (pA/pF)**	**Ctrl (pA/pF)**	**Cas 300nM (pA/pF)**
**-20mV**	-1.41±0.28	-0.70±0.17*	-21.97±6.85	-21.55±4.86	-12.28±3.31	-12.95±3.93
**-10mV**	-5.72±1.83	-1.76±0.46*	-24.04±6.83	-23.42±5.08	-15.68±3.76	-15.81±4.50
**0mV**	-9.25±2,32	-3.32±0.87*	-20.06±5.37	-19.52±4.21	-15.10±3.34	-14.76±4.28
**+10mV**	-9.90±1,94	-3.92±0.94*	-13.99±3.71	-13.16±2.99	-12.01±2.36	-11.09±3.45
**+20mV**	-8.05±1.41	-3.08±0.77**	-7.80±2.21	-6.44±1.89	-6.20±1.35	-6.12±2.78
**+30mV**	-4.66±0.82	-1.53±0.51**	-2.75±1.09	-1.13±1.30	-3.03±0.66	-2.96±2.36

L-type Ca^{2+} current density at different voltages in $Ca_v1.2$ and $Ca_v1.3$ transfected HEK-293T cells. *Control vs Cas 300nM. Statistics: unpaired student t -test. *p<0.05, **p<0.01. Data are presented as mean values ± SEM.

Supplementary Fig.4

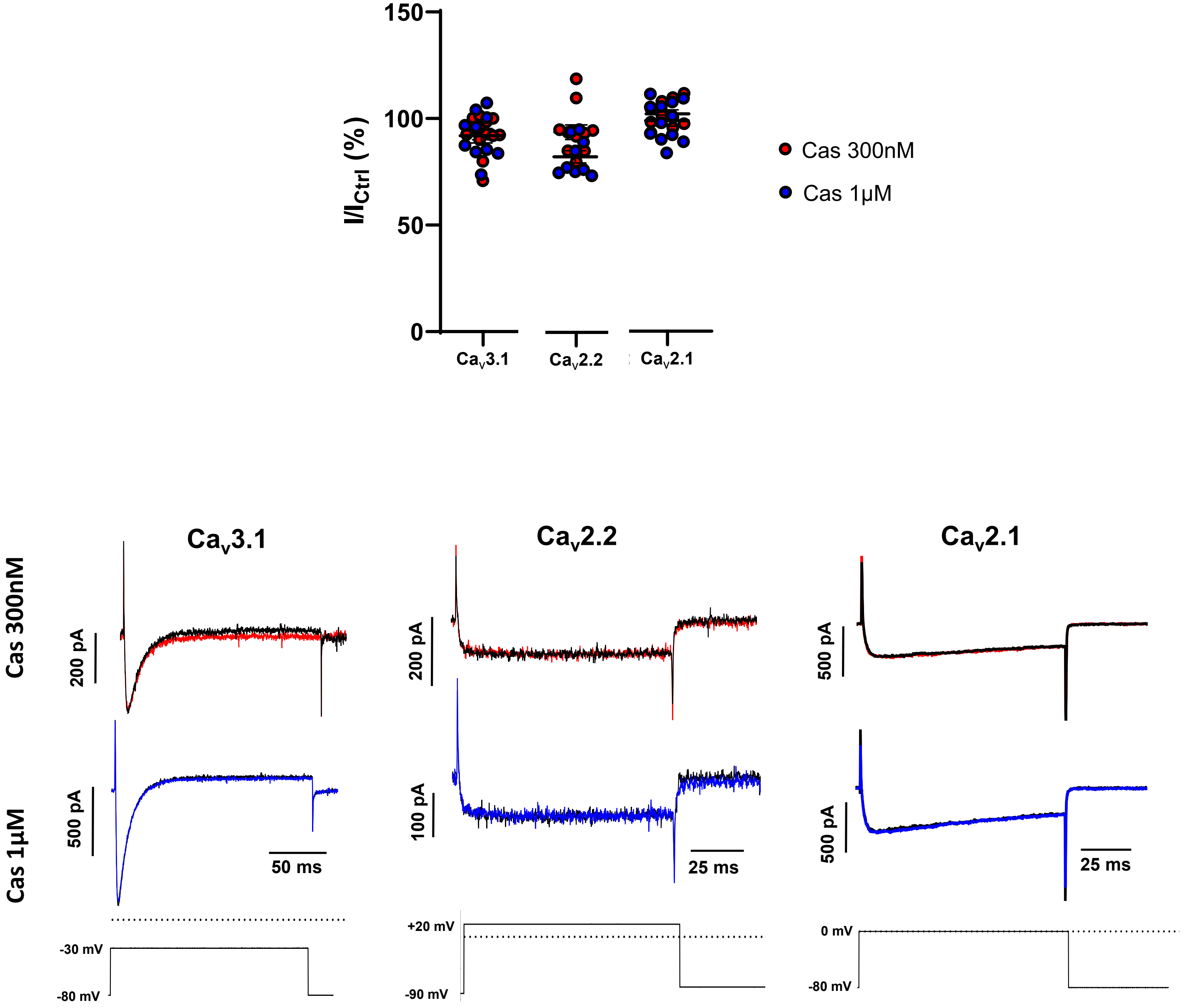

Cas effect on Ca^{2+}current density in HEK-293T cells expressing Ca$_v$3.1, Ca$_v$2.2, Ca$_v$2.1 channels. Dot plots represent the percentage of current density after Cas 300nM (red dots) and Cas 1µM (blue dots) in HEK-293T cells transfected with Ca$_v$3.1 (Cas 300nM, n=13/N=3; Cas 1µM n=10/N=3), Ca$_v$2.2 (Cas 300nM, n=11/N=3; Cas 1µM n=9/N=3) or Ca$_v$2.1 (Cas 300nM, n=10/N=3; Cas 1µM n=10/N=3) channels. Peak current density was recorded starting from a holding potential of -80mV using an activating voltage step at -30mV for Ca$_v$3.1, from a holding potential of -90mV using an activating voltage step at +20mV for Ca$_v$2.2 and from a holding potential of -80mV using an activating voltage step at 0mV for Ca$_v$2.1. Examples of recorded traces are reported. Statistics: one-way ANOVA test followed by Tukey's multiple comparisons test.

Data are presented as mean values ± SEM. n=number of cells; N=number of independent transfections.

Supplementary Fig.5

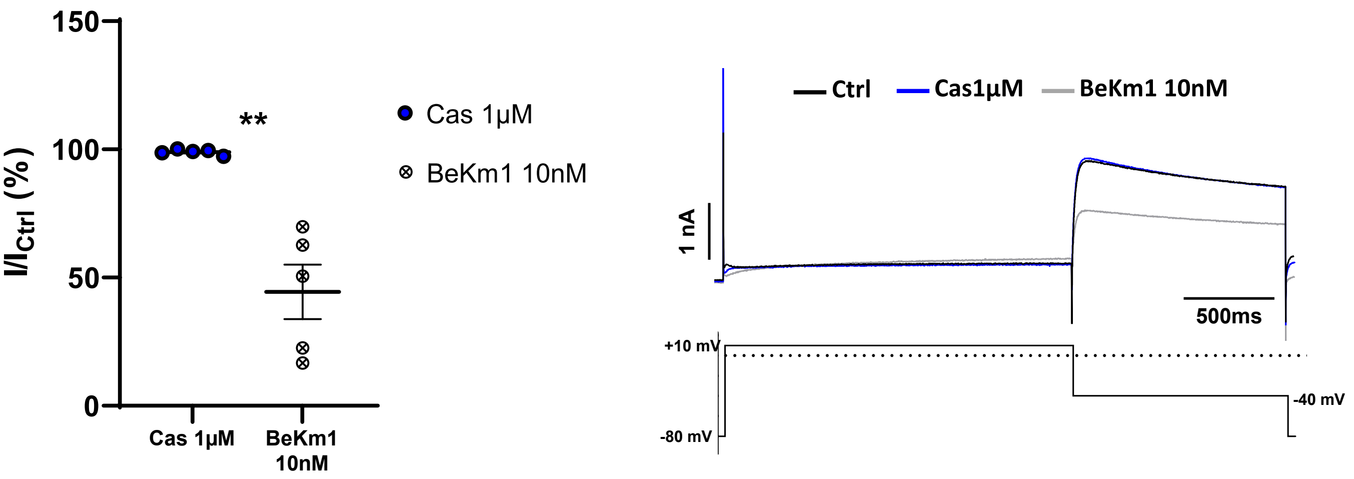

Left panel. Percentage (I/I_{ctrl}) of hERG current density after 1µM Cas perfusion and 10nM BeKm1 in oocytes transfected with hERG (n=5) channels. Right panel. Representative traces of hERG current before (black line), after 1µM Cas (blue line) and after 10nM BeKm1 perfusion. Recording protocol is showed under the traces. Statistics: Mann-Whitney test. $^{**}p<0.01$. Data are presented as mean values ± SEM.

Supplementary Fig.6

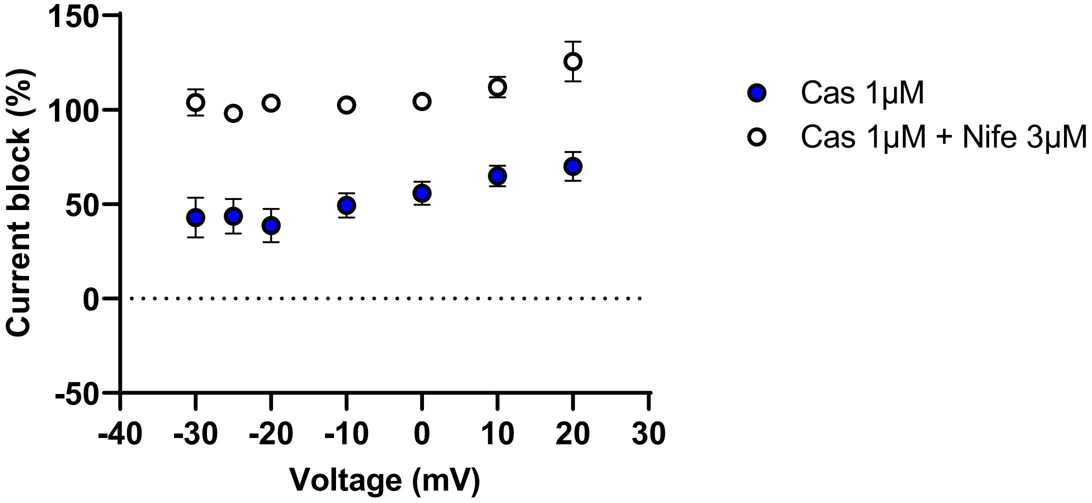

Percentage of current blocked by 1µM Cas (n=8, blue circles) or by 1µM Cas + 3µM Nifedipine (white circles) at membrane potential of -30mV, -25mV, -20mV, -10mV, 0mV, 10mV and 20mV in isolated WT SAN cells. Data are presented as mean values ± SEM.

	**Ctrl (pA/pF)**	**Cas 1µM (pA/pF)**	**Nife 3µM + Cas 1µM (pA/pF)**
**-35mV**	-1.18±0.29	-0.90±0.24$	-0.15±0.11#
**-30mV**	-2.44±0.47	-1.49±0.38$$	-0.01±0.10##
**-25mV**	-3.96±0.71	-2.46±0.48$	-0.08±0.12##
**-20mV**	-5.61±0.91*	-3.37±0.54$$	0.11±0.14####
**-10mV**	-8.20±1.10*	-4.24±0.77$$	0.08±0.14###
**0mV**	-8.45±1.02*	-3.87±0.71$$	0.17±0.19###
**+10mV**	-7.25±0,92**	-2,68±0.62$$$	0.51±0.27###
**+20mV**	-5.09±0.81**	-1.53±0.56$$	0.73±0.35###

L-type Ca^{2+} current density at different voltages in isolated SAN wild-type myocytes. *Control vs Cas 1µM, #Control vs Nife 3µM + Cas 1µM, $Cas 1µM vs Nife 3µM + Cas 1µM. Statistics: one-way ANOVA test followed by Tukey's multiple comparisons test. *p<0.05, **p<0.01, $p<0.05, $$p<0.01, $$$p<0.001, #p<0.05, ##p<0.01, ###p<0.001 ####p<0.0001. Data are presented as mean values ± SEM.

Supplementary Fig.7

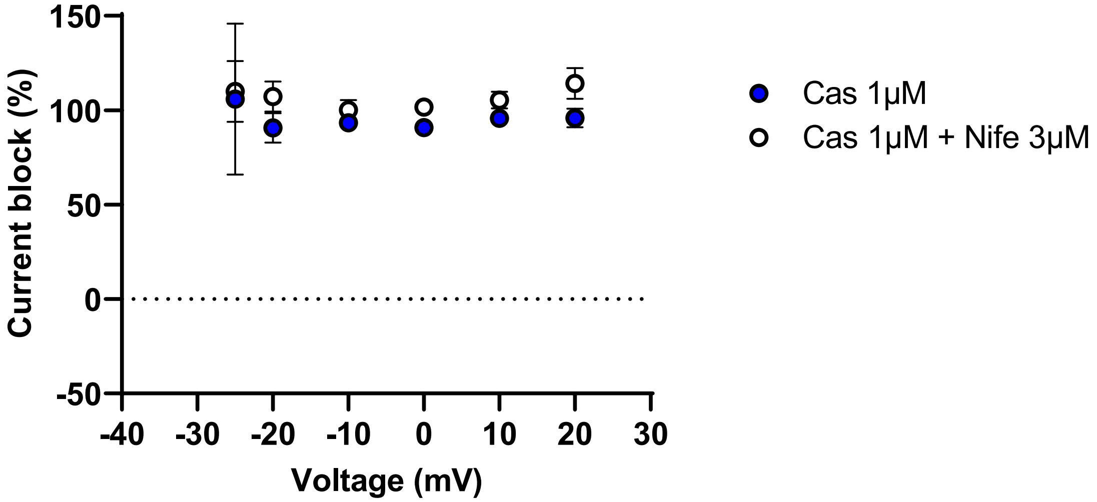

Percentage of current blocked by 1µM Cas (n=8, blue circles) or by 1µM Cas + 3µM Nifedipine (white circles) at membrane potential of -25mV, -20mV, -10mV, 0mV, 10mV and 20mV in isolated $Ca_v1.3^{-/-}$ SAN cells. Data are presented as mean values ± SEM.

	Ctrl (pA/pF)	Cas 1µM (pA/pF)	Nife 3µM + Cas 1µM (pA/pF)
**-25mV**	-0.97±0.21*	-0.25±0.13	-0.04±0.10$^{###}$
**-20mV**	-1.16±0.20*	-0.08±0.03	0.14±0.09$^{#}$
**-10mV**	-2.49±0.39**	-0.17±0.07	0.13±0.14$^{##}$
**0mV**	-4.14±0.56**	-0.45±0.14	0.07±0.13$^{##}$
**+10mV**	-4.69±0.57**	-0.17±0.14	0.31±0.23$^{##}$
**+20mV**	-3.93±0.56**	-0.13±0.21	0.40±0.24$^{##}$
**+40mV**	-1.15±0.37**	0.37±0.29	0.95±0.50$^{##}$

L-type Ca^{2+} current density at different voltages in isolated SAN $Ca_v1.3^{-/-}$ myocytes. *Control vs Cas 1µM, $^{#}$Control vs Nife 3µM + Cas 1µM. Statistics: one-way ANOVA test followed by Tukey's multiple comparisons test. *p<0.05, **p<0.01, $^{#}$p<0.05, $^{##}$p<0.01, $^{###}$p<0.001. Data are presented as mean values ± SEM.

Supplementary Fig.8

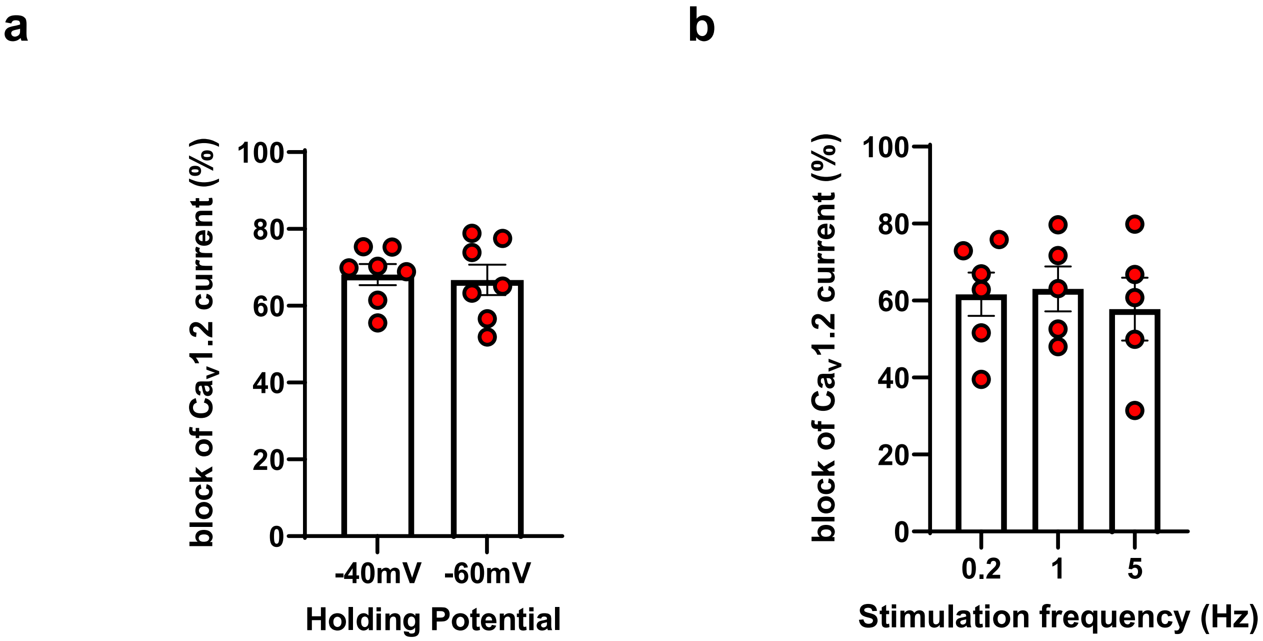

a. Percentage of $Ca_v1.2$-mediated current inhibited after Cas 300nM perfusion in isolated SAN myocytes (n=7/N=2) from $Ca_v1.3^{-/-}$ mice recorded using voltage clamp 0mV step protocol with an holding potential of -40mV or -60mV. Statistic: unpaired Student t-test. **b**. Percentage of $Ca_v1.2$-mediated current inhibited after Cas 0.3μM perfusion in isolated SAN myocytes (n=6/N=2) from $Ca_v1.3^{-/-}$ mice recorded using voltage clamp 0mV step protocol, holding potential of -60 mV, with different repetition frequency (0.2, 1 and 5Hz). Statistic: one-way ANOVA followed by Tukey multiple comparisons test.

Data are presented as mean values ± SEM. n=number of cells; N=number of mice.

Supplementary Fig.9

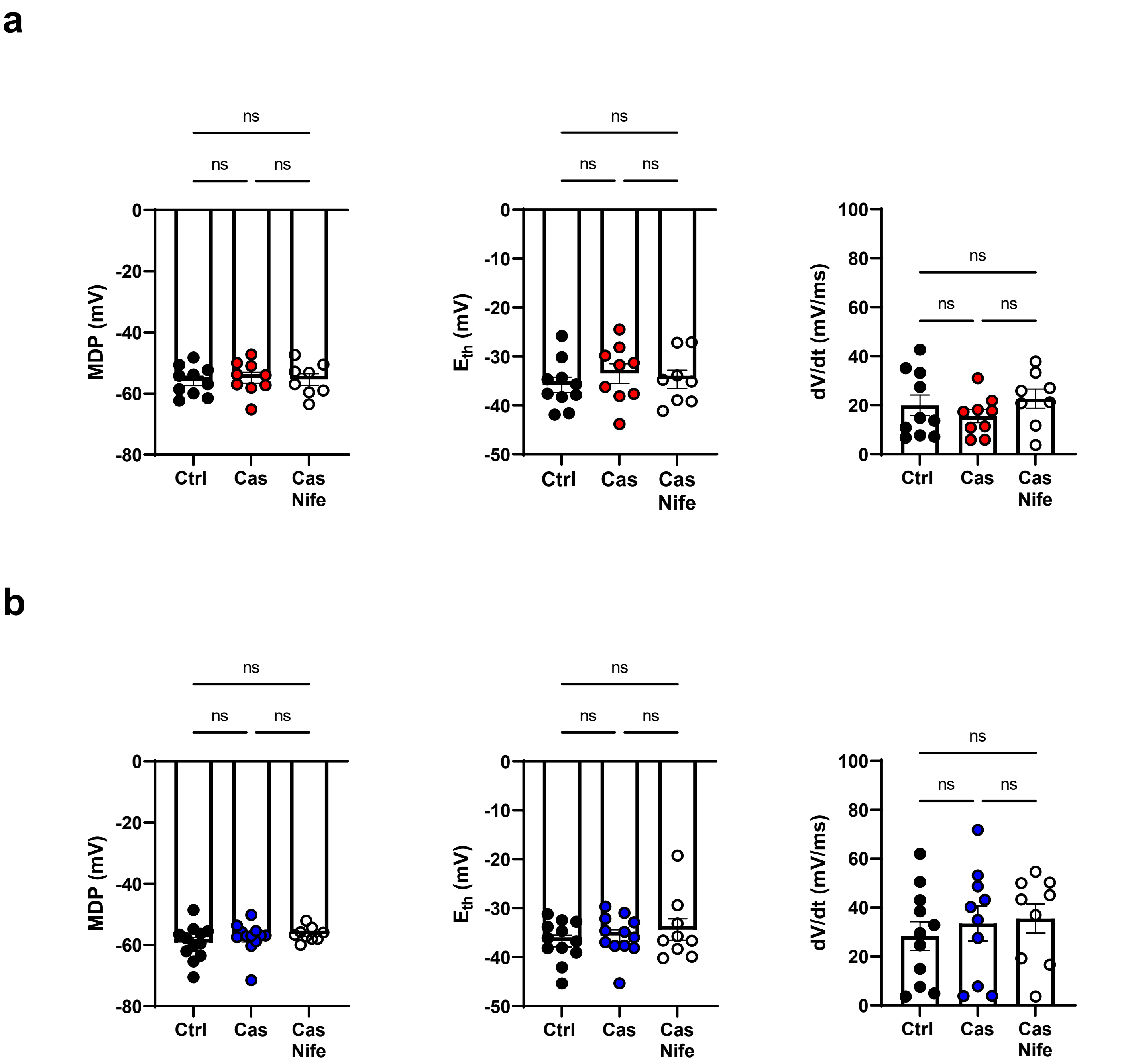

a. Histograms of maximum diastolic potential (MDP), threshold potential (E_{th}) and upstroke velocity (dV/dt) of action potential measured in SAN myocytes in basal conditions (n=10/N=4, Ctrl), after perfusion with Cas 300nM (n=9/N=3, Cas, red filled circles) and under concomitant application of Cas 300nM+Nife 3μM (n=8/N=4, empty circles). **b**. Same as **a** but with Cas 1μM (n=12/N=3, Ctrl; n=12/N=3 Cas1μM and n=10/N=3 Cas 1μM+Nife 3μM). Statistic: one-way ANOVA test followed by Tukey's multiple comparisons test. Data are presented as mean values ± SEM. n=number of cells; N=number of mice.

Supplementary Fig.10

a

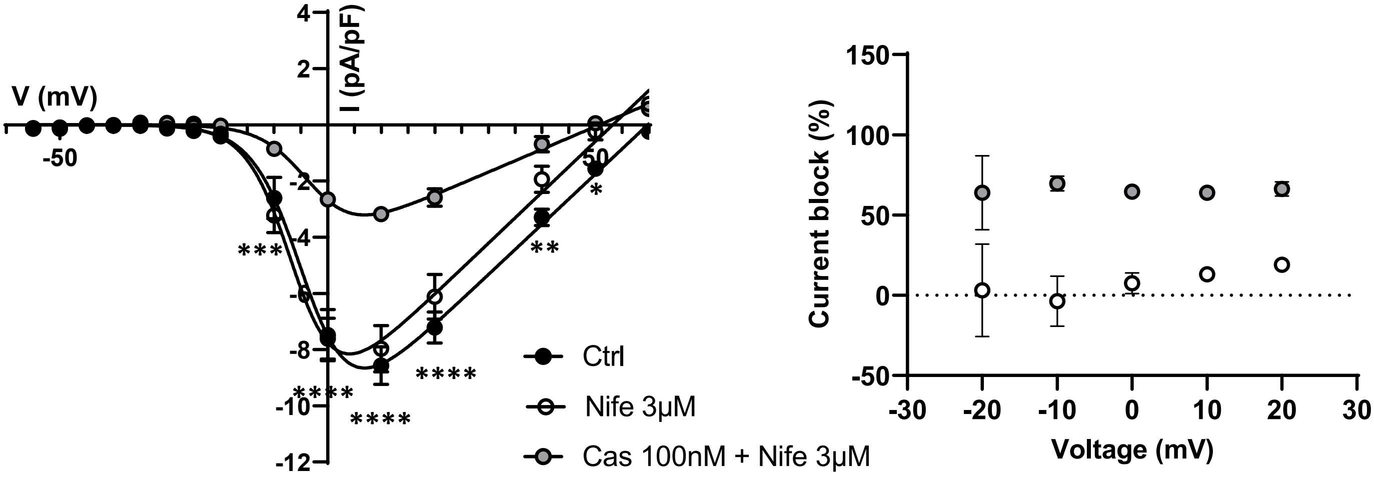

(Left panel). L-type Ca^{2+} current density at different voltages in isolated ventricular $Ca_v1.2^{DHP-/-}$ myocytes (n=9/N=2) before (black filled circles) after perfusion with 3μM Nife (empty circles) and after concomitant perfusion of native 100nM Cas and 3μM Nife (gray filled circles). * Nife 3μM vs Cas 100nM + Nife 3μM Statistics: two-way ANOVA followed by Tukey's multiple comparisons test. *p<0.05, **p<0.01, ***p<0.001, ****p<0.0001. (Right panel). Percentage of current blocked by 3μM Nife (empty circles) and by 100nM Cas + 3μM Nife (gray circles) at membrane potential of -20mV, -10mV, 0mV, 10mV and 20mV in isolated $Ca_v1.2^{DHP-/-}$ ventricular cells. Data are presented as mean values ± SEM. n=number of cells; N=number of mice.

b

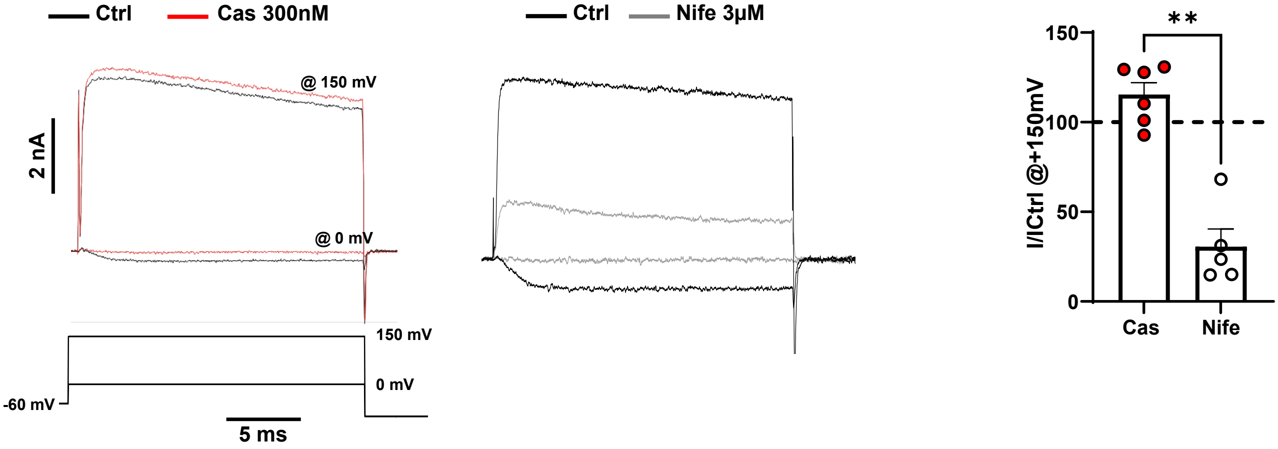

Cas effect on $Ca_v1.2$ Ca^{2+} current alternatively elicited at 0 mV and at +150 mV in HEK-293T cells expressing Cav1.2 channels (inter-pulse 10s). Histograms represent L-type Ca^{2+} normalized (to control) current density evoked by step potential at 0 mV and 150 mV from an holding potential of -60mV under perfusion of Cas 300nM (n=6/N=3, red line) or Nife 3μM (n=5/N=2 gray line). Statistics: Mann-Whitney test **p<0.01. Data are presented as mean values ± SEM. n=number of cells; N=number of independent transfections.

Supplementary Fig.11

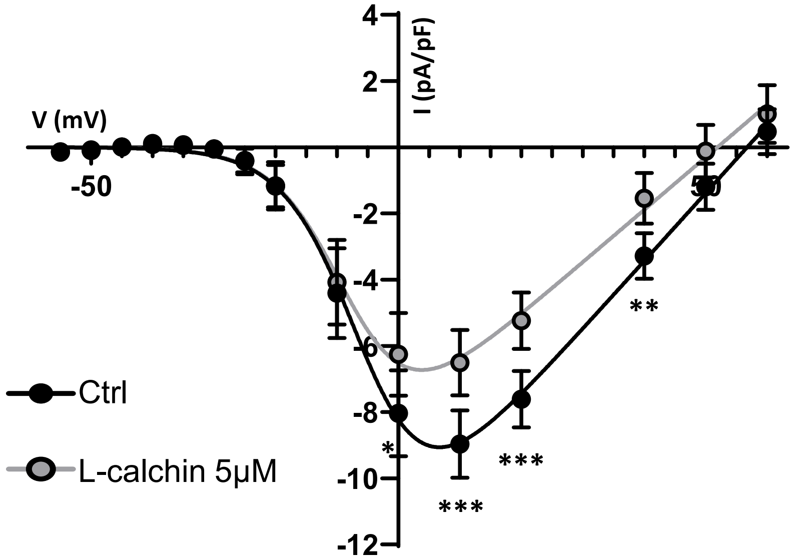

L-type Ca^{2+} current density at different voltages in isolated ventricular Wild-Type myocytes (n=7/N=2) before (black filled circles) and after 5μM L-calchin perfusion (gray filled circles). Statistics: paired Student t-test. *p<0.05, **p<0.01, ***p<0.001. Data are presented as mean values ± SEM. n=number of cells; N=number of mice

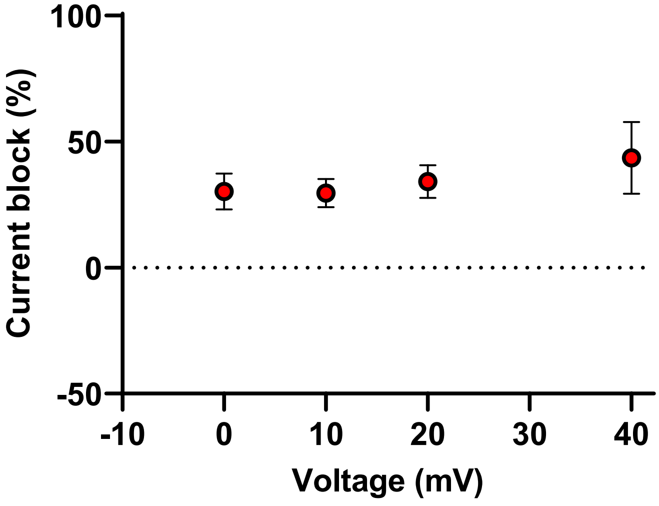

Percentage of current blocked by 5μM L-calchin (red circles) at membrane potential of 0mV, 10mV, 20mV and 40mV in isolated WT ventricular cells. Data are presented as mean values ± SEM.

Supplementary Fig.12
