# Supplementary material for: Selective blockade of Cav1.2 (α1C) versus Cav1.3 (α1D) L-type calcium channels by the black mamba toxin calciseptine
Source: Nat Commun. 2024 Jan 2;15:54. doi: 10.1038/s41467-023-43502-w (PMC10762068; doi:10.1038/s41467-023-43502-w)
Supplement: Supplementary file 3 — Reporting Summary [file 41467_2023_43502_MOESM3_ESM.pdf]

nature portfolio

Corresponding author(s): Pietro Mesirca, Joël Nargeot

Last updated by author(s): Oct 20, 2023

Reporting Summary

Nature Portfolio wishes to improve the reproducibility of the work that we publish. This form provides structure for consistency and transparency in reporting. For further information on Nature Portfolio policies, see our Editorial Policies and the Editorial Policy Checklist.

Statistics

For all statistical analyses, confirm that the following items are present in the figure legend, table legend, main text, or Methods section.

n/a	Confirmed	
☐	☒	The exact sample size (*n*) for each experimental group/condition, given as a discrete number and unit of measurement
☐	☒	A statement on whether measurements were taken from distinct samples or whether the same sample was measured repeatedly
☐	☒	The statistical test(s) used AND whether they are one- or two-sided<br>*Only common tests should be described solely by name; describe more complex techniques in the Methods section.*
☒	☐	A description of all covariates tested
☒	☐	A description of any assumptions or corrections, such as tests of normality and adjustment for multiple comparisons
☐	☒	A full description of the statistical parameters including central tendency (e.g. means) or other basic estimates (e.g. regression coefficient) AND variation (e.g. standard deviation) or associated estimates of uncertainty (e.g. confidence intervals)
☐	☒	For null hypothesis testing, the test statistic (e.g. *F*, *t*, *r*) with confidence intervals, effect sizes, degrees of freedom and *P* value noted<br>*Give P values as exact values whenever suitable.*
☒	☐	For Bayesian analysis, information on the choice of priors and Markov chain Monte Carlo settings
☒	☐	For hierarchical and complex designs, identification of the appropriate level for tests and full reporting of outcomes
☒	☐	Estimates of effect sizes (e.g. Cohen's *d*, Pearson's *r*), indicating how they were calculated

Our web collection on statistics for biologists contains articles on many of the points above.

Software and code

Policy information about availability of computer code

Data collection	IOX data acquisition system (EMKA Technologies, France). Axopatch 200B or Multiclamp 700B patchclamp amplifier (Molecular Devices). Clampfit 10.7.0.3 (Molecular Devices).
Data analysis	ecgAUTOv3.3.3.12 (EMKA technologies); MATLAB-R2021b (The MathWorks). Statistical analysis was performed using Prism 10.0 (GraphPad Software)

For manuscripts utilizing custom algorithms or software that are central to the research but not yet described in published literature, software must be made available to editors and reviewers. We strongly encourage code deposition in a community repository (e.g. GitHub). See the Nature Portfolio guidelines for submitting code & software for further information.

Data

Policy information about availability of data

All manuscripts must include a data availability statement. This statement should provide the following information, where applicable:

- Accession codes, unique identifiers, or web links for publicly available datasets
- A description of any restrictions on data availability
- For clinical datasets or third party data, please ensure that the statement adheres to our policy

All data that support the findings of this study are included in the manuscript. Original files of electrophysiological recordings and raw data are available upon request to the corresponding authors

Research involving human participants, their data, or biological material

Policy information about studies with human participants or human data. See also policy information about sex, gender (identity/presentation), and sexual orientation and race, ethnicity and racism.

Reporting on sex and gender	N/A
Reporting on race, ethnicity, or other socially relevant groupings	N/A
Population characteristics	N/A
Recruitment	N/A
Ethics oversight	N/A

Note that full information on the approval of the study protocol must also be provided in the manuscript.

Field-specific reporting

Please select the one below that is the best fit for your research. If you are not sure, read the appropriate sections before making your selection.

[x] Life sciences [ ] Behavioural & social sciences [ ] Ecological, evolutionary & environmental sciences

For a reference copy of the document with all sections, see nature.com/documents/nr-reporting-summary-flat.pdf

Life sciences study design

All studies must disclose on these points even when the disclosure is negative.

Sample size	We did not perform a priori sample size calculations. We have estimated the number of cells required in line with our experience on native calcium channels in cardiac myocytes, as well as heterologously expressed channels in HEK cells. We based samples calculations on native myocytes from sinus node and ventricle upon the following manuscripts: Mangoni et al. PNAS 2003 (PMID: 12700358), Baudot et al. Sci Rep 2020 (PMID: 33144668), De Waard et al. 2020 Biochem J (PMID: 33034621). This bulk of previous evidence ensures that differences in amount of current blockade as low as 10% can be evidenced with the number of native myocytes used in the present study. As to heterologously expressed channels, we took as reference the following works: Monteil et al. JBC 2000a and b (PMID: 10749850 and PMID: 10692398). The number of cells we tested in the present work is in line with the number of cells that were investigated and recorded in these previous works.
Data exclusions	No data were excluded from the analyses.
Replication	Experiments reported in the present study were evenly performed over a 1.5-year period. Each experiment has been performed several times over the considered period and results reflect the sum of all experimental rounds. We also confirm that all replication attempts were successful.
Randomization	Samples were allocated randomly into experimental groups.
Blinding	It was not relevant to blind experimental protocol for this specific study. Indeed, sinoatrial pacemaker myocytes are visually too different to be confounded with ventricular myocytes. Cav1.3 knockout cells have very low pacemaking visually, therefore they are visually distiguished from wild-type counterparts. Cav-transfected HEK cells expres GFP, they are thus visually distinguished from non-transfected counterparts. Effects of the toxin is evaluated by current reduction (or not) on live experiments , and all data irrespective of the amount of effect were included. For all these reseasons, blinding strategy of experimantal protocol did not apply to our study.

Reporting for specific materials, systems and methods

We require information from authors about some types of materials, experimental systems and methods used in many studies. Here, indicate whether each material, system or method listed is relevant to your study. If you are not sure if a list item applies to your research, read the appropriate section before selecting a response.

Materials & experimental systems

n/a	Involved in the study	
☒	☐	Antibodies
☐	☒	Eukaryotic cell lines
☒	☐	Palaeontology and archaeology
☐	☒	Animals and other organisms
☒	☐	Clinical data
☒	☐	Dual use research of concern
☒	☐	Plants

Methods

n/a	Involved in the study	
☒	☐	ChIP-seq
☒	☐	Flow cytometry
☒	☐	MRI-based neuroimaging

Eukaryotic cell lines

Policy information about cell lines and Sex and Gender in Research

Cell line source(s)	HEK-293T (tsA-201) cells (RRID:CVCL_2737) were obtained from the European Collection of Authenticated Cell Cultures (ECACC 96121229). The identity of HEK-293T has been confirmed by STR profiling and the cells have been eradicated from mycoplasma at ECACC.
Authentication	HEK-293T (tsA-201) cells (RRID:CVCL_2737) were obtained from the European Collection of Authenticated Cell Cultures (ECACC 96121229). The identity of HEK-293T has been confirmed by STR profiling and the cells have been eradicated from mycoplasma at ECACC. We routinely tested the cells for the absence of the mycoplasma contamination.
Mycoplasma contamination	HEK293-T cells have been eradicated from mycoplasma at ECACC. We routinely tested the cells for the absence of the mycoplasma contamination.
Commonly misidentified lines (See ICLAC register)	N/A

Animals and other research organisms

Policy information about studies involving animals; ARRIVE guidelines recommended for reporting animal research, and Sex and Gender in Research

Laboratory animals	WT, Cavl.2DHP-/- and Cavl.3-/- mice (2 to 4 months old) used in this study were bred on C57B1/6J background.
Wild animals	This study did not involve wild animais.
Reporting on sex	Sex was not considered in this study.
Field-collected samples	This study did not involve samples collected from the field.
Ethics oversight	The study is in accordance with the Guide for the Care and Use of Laboratory Animais published by the US National Institute of Health (NIH Publication No. 85-23, revised 1996) and European directives (2010/63/EU). Experimental procedures were approved by the Ethical Committee of the University of Montpellier and the French Ministry of Agriculture (protocol no: 2017010310594939).

Note that full information on the approval of the study protocol must also be provided in the manuscript.
